# Patient education for people with multiple sclerosis-associated fatigue: A systematic review

**DOI:** 10.1371/journal.pone.0173025

**Published:** 2017-03-07

**Authors:** Maria Janina Wendebourg, Christoph Heesen, Marcia Finlayson, Björn Meyer, Jana Pöttgen, Sascha Köpke

**Affiliations:** 1 Institute of Neuroimmunology and Department of Neurology, University Medical Center, Hamburg-Eppendorf, Hamburg, Germany; 2 School of Rehabilitation Therapy, Queen’s University, Kingston, Ontario, Canada; 3 GAIA AG, Hamburg, Germany; 4 Nursing Research Unit, Institute of Social Medicine and Epidemiology, University of Lübeck, Lübeck, Germany; Hannover Medical School, GERMANY

## Abstract

**Background:**

Multiple Sclerosis (MS) is an inflammatory and neurodegenerative disease often causing decreased quality of life, social withdrawal and unemployment. Studies examining the effect of pharmacological interventions demonstrated only minor effects, whereas non-pharmacological interventions as e.g. patient education programs have shown promising results.

**Objective:**

We aim to systematically review the literature to determine the effect of patient education programs on fatigue in MS.

**Methods:**

We conducted a comprehensive search in PubMed for randomized controlled trials (RCTs) that evaluated patient education programs for MS-related fatigue. Interventions evaluating physical exercise and/or pharmacological treatments were not included. Meta-analyses were performed using the generic inverse variance method.

**Results:**

The search identified 856 citations. After full-text screening we identified ten trials that met the inclusion criteria. Data of 1021 participants were analyzed. Meta-analyses showed significant positive effects on fatigue severity (weighted mean difference -0.43; 95% CI -0.74 to -0.11) and fatigue impact (-0.48; -0.82 to -0.15), but not for depression (-0.35 (95% CI -0.75 to 0.05; p = 0.08). Essentially, we categorized patient education programs into two types: firstly, interventions with a focus on cognitive-behavioral therapy (CBT) and secondly, interventions that teach patients ways of managing daily fatigue. CBT-based approaches seem to generate better results in reducing patient-reported fatigue severity. Analysing CBT studies only, the pooled weighted mean difference for fatigue severity was -0.60 (95% CI; -1.08 to -0.11) compared to non-CBT approaches (-0.20; 95% CI; -0.60 to -0.19). Furthermore, interventions employing an individual approach seem to reduce fatigue more effectively than group-based approaches (pooled weighted mean difference for fatigue severity in face-to-face studies was -0.80 (95% CI; -1.13 to -0.47) compared to group-based studies with -0,17 (95% CI; -0,39 to 0,05). Longest follow-up data were available for 12 months post-intervention.

**Conclusion:**

Overall, included studies demonstrated that educational programs and especially CBT-based approaches have a positive effect on reducing fatigue. Since fatigue is thought to be a multidimensional symptom, it should be treated with a multidimensional approach targeting patients’ behavior as well as their emotional and mental attitude towards fatigue. However, the clinical relevance of the treatment effects i.e. the relevance for patients’ daily functioning remains unclear and long-term effects, i.e. sustainability of effects beyond 6 months, warrants further work. This review has been registered in the PROSPERO international prospective register of systematic reviews data base (Registration number: CRD42014014224).

## Introduction

Multiple Sclerosis (MS) is a poorly understood disease of the central nervous system that mostly starts in young adulthood [1]. Disease progression is unpredictable and patients may experience a wide range of symptoms. Amongst limb weakness, paraesthesia, visual impairment and other symptoms patients regularly report fatigue. Fatigue is one of the most common symptoms that 75–95% of MS-patients struggle with at least once in their life [2]. Current definitions include a subjective lack of physical and/or mental energy that is perceived by the individual or the caregiver to interfere with usual or desired activity, an overwhelming sense of tiredness, lack of energy or feelings of exhaustion and a feeling of physical tiredness and lack of energy distinct from sadness or weakness [3]. Fatigue is considered to be the most disabling symptom by many patients, more important than mobility restrictions or pain [4]. Fatigue is also a common reason for unemployment [5] and often a cause for social withdrawal [6]. Fatigue may affect patients very differently and causes are yet poorly understood [3].

According to current beliefs, there are primary and secondary mechanisms that lead to fatigue [7]. While primary fatigue is a direct result of physical changes within the body through the disease itself, secondary fatigue is due to other primary factors that can cause and worsen fatigue. Among the primary mechanisms are axonal loss, reorganization and increased brain recruitment as well as immunological and neuroendocrine factors. Factors of secondary fatigue include sleeping problems, depression, stress, side effects of pharmacological therapies and reduced physical activity [8; 9]. Moreover, it has been postulated that fatigue is perpetuated by certain cognitions and beliefs [10].

Currently, the only established way of measuring fatigue is by use of self-reported scales since fatigue is subjective and multidimensional symptom. Most common are scales that measure fatigue severity i.e. the intensity and characteristics of experienced fatigue and/or fatigue impact which focuses on activities that patients can and cannot do due to fatigue. These two measurements do not always correspond because perceived intensities may not have the same impact on different patients’ life [11].

Currently there are no convincing pharmacological treatments available for MS-related fatigue [12]. However, there have been a number of non-pharmacological treatment approaches. For example, physical exercise seems to have positive results on fatigue severity [13].

A current systematic review and meta-analysis has suggested promising results of various patient education programs teaching patients fatigue management techniques [14]. The review compared the effects of medication, physical exercise and patient education programs for MS-related fatigue and concluded that physical exercise and education were more effective than pharmacological therapies and should therefore be preferred in fatigue therapy management. The review however only gives a broad overview on different interventions.

As there are various approaches on educating patients and teaching them different skills to better manage and cope with fatigue in daily life, a more focussed review was considered necessary. For this review, we aimed to include two different types of programs: Firstly, programs based on cognitive-behaviour therapy and secondly, programs teaching patients’ general strategies of fatigue management. We aimed to describe the complex interventions in detail, using a recently proposed framework and to analyse the quality of the evidence using the Cochrane risk of bias tool [15] and GRADE methodology [16]. Furthermore, we recorded length and duration of each program as well as settings and implementation strategies. In summary we aimed to determine the best strategy for patient education programs to achieve best results in reducing fatigue.

The objective of the current review is to summarize the results of RCTs assessing the effects of CBT-based patient education compared to other patient education programs that teach ways of managing fatigue in daily life for the reduction of MS-related fatigue in any setting.

## Methods

This review is based on systematic literature searches in PubMed and the Cochrane library. As we were only looking for RCTs with at least 30 participants, we decided to perform a sensitive search in PubMed as this has been shown to be highly sensitive [17]^.^ Additionally, we searched the Cochrane library and checked reference lists of included studies and other systematic reviews to increase sensitivity. The latest search was conducted on the 3^rd^ January, 2016. We aimed for a maximum sensitive search and therefore only used the search terms “multiple sclerosis” and “fatigue”. The explicit search strategy can be found in the appendix.

### Inclusion criteria

Only studies that evaluated patient education programs for MS-related fatigue using randomized controlled and crossover designs were included. As education program we defined any intervention where patients receive information or education about strategies to deal with fatigue. Further inclusion criteria were fatigue as a primary outcome measure and a minimum effective sample size of 30 participants. Additionally, we only included studies that assessed fatigue at least at two time points. Studies that included patients with other neurodegenerative/neuroinflammatory diseases than MS were included if the majority of included patients were MS patients. No limitations concerning publication date, disease course of participants, or language were made.

Eligibility for studies was assessed independently by two reviewers. Discrepancies were solved by discussion afterwards. Reviewers were not blinded to study authors. Data extraction was performed according to a standardized data extraction form. Data for the following items was extracted: (1) sample size, (2) demographic data of participants, (3) type and length of intervention (number of weeks, number of hours per week), (4) type of control group, (5) primary and secondary outcomes, (6) length of follow-up data collection, and (7) baseline, post-intervention and, if applicable, follow-up data for fatigue, depression and quality of life.

### Risk of bias assessment

We used the Cochrane Risk of Bias tool to assess study quality. This tool evaluates selection bias, performance bias, detection bias, attrition bias, and reporting bias. Due to the nature of the included studies, it is difficult to blind participants to group allocation since patient education programs require participants to play an active part in the sessions [18]. In combination with all outcome measures being entirely self-reported, this was regarded as a potentially high risk of bias.

### Summary measures

All studies were assessed by two independent reviewers. As all outcomes were continuous outcomes, we calculated standardised mean differences (SMD) between groups and 95% confidence interval when sufficient data were available. We contacted authors of studies in case of insufficient information on data at baseline and follow-up. For meta-analyses, we used the generic inverse variance approach using RevMan software [19]. Due to marked clinical heterogeneity, we used random effects models to provide pooled estimated effects.

We additionally performed subgroup analyses by calculating pooled mean differences for trials that worked with a CBT approach and individual vs. group interventions. We define the term ‘individual intervention’ as the session being based on one therapist and one patient in direct contact (face-to-face, via telephone or online).

### Risk of bias across studies

We used the specific evidence grading system developed by the GRADE [16] group to assess study quality.

## Results

### Study selection (see Fig 1)

856 publications were identified by the search. After deleting 78 duplicates, 778 studies remained to be screened by abstract which ultimately led to 30 publications for full-text screening. After reading full-text articles, 20 publications were eliminated for different reasons, namely trials using drugs, sports interventions, magnetic fields, complementary medicine and others. 10 publications that met all criteria were included. For one study we thankfully received additional data from the authors which enabled us to integrate the RCT into our analyses.

**Fig 1:**
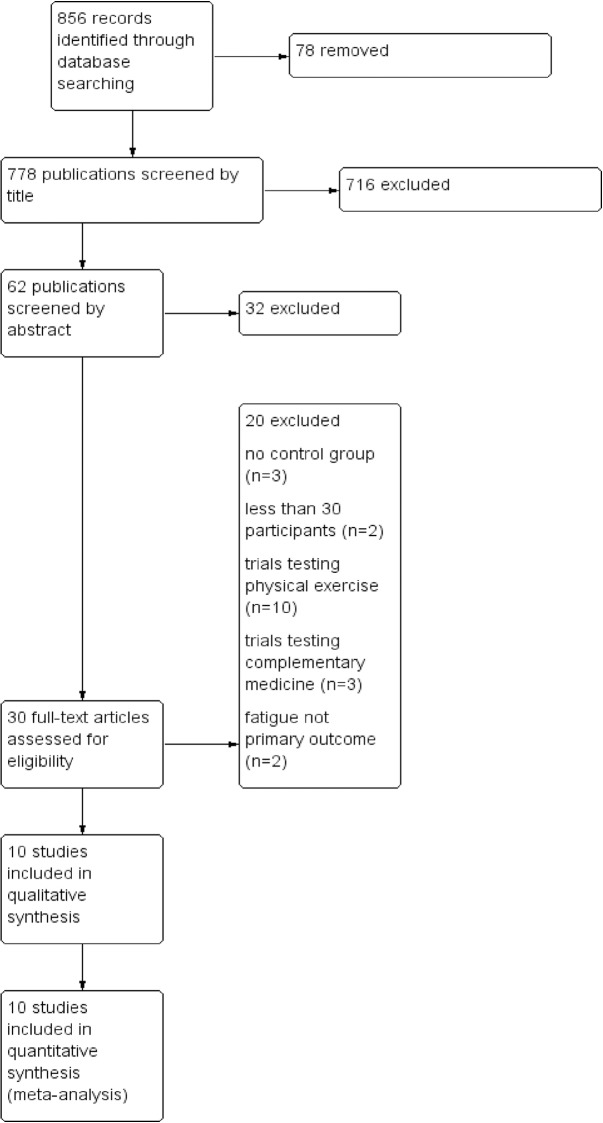
Study selection process.

### Study characteristics (see Table 1)

All included studies were randomized controlled trials published in English, two of which used a crossover design. According to the information provided in the respective publications, group and face-to-face sessions took place in community centres or hotel conference rooms. No interventions were performed in a hospital setting. Interventions took four to ten weeks and follow-up data were available from ten weeks to 12 months. Control groups contained usual care, “placebo” interventions and alternative interventions e.g., relaxation training and physical exercise. Trials were performed in the US (n = 4), Belgium (n = 1), Australia (n = 1), the UK (n = 2), Switzerland (n = 1) and New Zealand (n = 1).

**Table 1:** Overview of types of interventions, sample sizes, dates, countries and methods of delivery.

	Country	Date	SampleSize	Intervention	Method of delivery
Finlayson et al. 2011[20]	USA	11/2007-04/2009	190	Managing fatigue program	Group teleconference
Ghahari et al. 2010[21]	Australia	05/2007-03/2008	95	Managing fatigue program	Online + online personal contract with facilitators
Grossman et al. 2010[22]	Switzerland	n.r.	150	Mindfulness-based intervention	Group sessions
Hugos et al. 2009[23]	USA	03-08/2007	30	Fatigue self-management program	Group setting
Kos et. Al. 2007[9]	Belgium	n.r.	51	Multidisciplinary fatigue management program	Group setting
Mathiowetz et al. 2005[24]	USA	USA, 2002–3	169	Managing fatigue program	Group setting
Moss-Morris et al. 2012[25]	UK	n.r.	40	Cognitive-behavioral therapy	Online + telephone contact with facilitators
Thomas et al. 2013[26]	UK	11/2007-04/2009	164	Cognitive-behavioral therapy + energy effectiveness training	Group sessions
Van Kessel et al. 2008[27]	New Zealand	07/2004-08/2005	72	Cognitive-behavioral therapy	Personal contact + telephone sessions
Mohr et al. 2003[28]	USA	n.r.	60	Cognitive-behavioral therapy	Personal contact sessions

n.r. = not reported

### Participants

The number of study participants ranged from 30 to 190. Overall, this review contains data from 1021 participants from 10 trials. In all studies, predominantly women participated which corresponds to the male: female ratio of patients in MS patients. Mean age of participants ranged from 41.1 to 56 years. Relapsing-remitting MS (RRMS) was the predominantly reported disease course in all studies. Mean years since disease onset ranged from 5.5 to 21 years.

### Interventions (see Fig 2)

Cognitive-behavioral therapy was the most commonly used approach. Four of ten approaches were based on CBT. Two studies used an energy conservation program. Three studies used multidisciplinary fatigue self-management programs that informed about different techniques of reducing fatigue e.g. relaxation, cooling, energy saving but also teaching the benefit of physical exercise. One study employed the concept of mindfulness.

**Fig 2:**
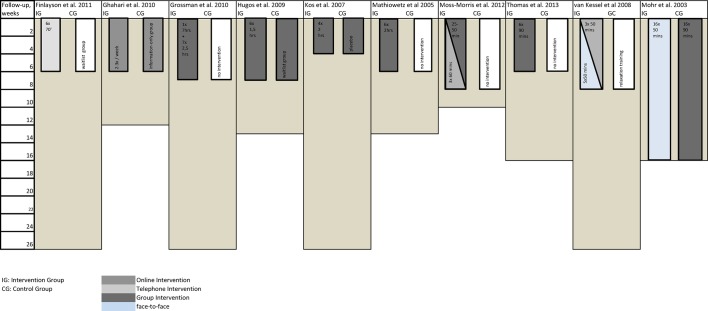
Overview of study length, type of intervention and follow-up.

Different ways of program delivery were employed. Mainly, two different formats are distinguishable: group programs and individual programs. Group sessions were the most commonly used format. Further, there were different ways of contact between therapist and participant, e.g. personal contact, online and telephone contact. Two interventions used online sessions; one with online personal contact to the facilitators, one combined online with telephone support sessions.

Another difference is the length of intervention in weeks (and weekly hours) as well as length of follow-up. The shortest intervention was 4 weeks and the longest was 16 weeks. Fig 2 gives an overview of the types of program, length of the program and follow-up.

### Outcomes

Interventions used different fatigue measures. The most commonly administered outcome measures were the Fatigue Impact Scale [29] and the Modified Fatigue Impact Scale [30], each used in four trials. Further outcome measures were the Fatigue Severity Scale [31] and the Fatigue Scale [32]. Furthermore, depression was assessed in 6 studies and health-related quality of life in 3 studies. Also, some studies assessed anxiety as secondary outcome measurements. All outcomes measures were taken at baseline and directly post-intervention. 6 studies reported follow up data for fatigue impact and 3 studies for fatigue severity (between three and six months).

### Risk of bias in individual studies (Fig 3)

We did not detect selection bias in most studies. Due to the nature of patient education interventions, staff and patient blinding was not realized as participants and facilitators engage with each other, but this was considered as low risk of bias. To control for therapist time and attention, van Kessel and colleagues used relaxation training [27]. Outcome assessment was considered as blinded in all studies although all outcomes were self-reported. We assessed outcome data as incomplete if more than 10% of participants’ data was not missing. This was the case in one study. To assess selective reporting we searched for published study protocols. If those were not found, we judged the risk as ‘unclear’ (this applied to 6 studies). Furthermore, we assessed study quality with the GRADEpro tool [16].

**Fig 3:**
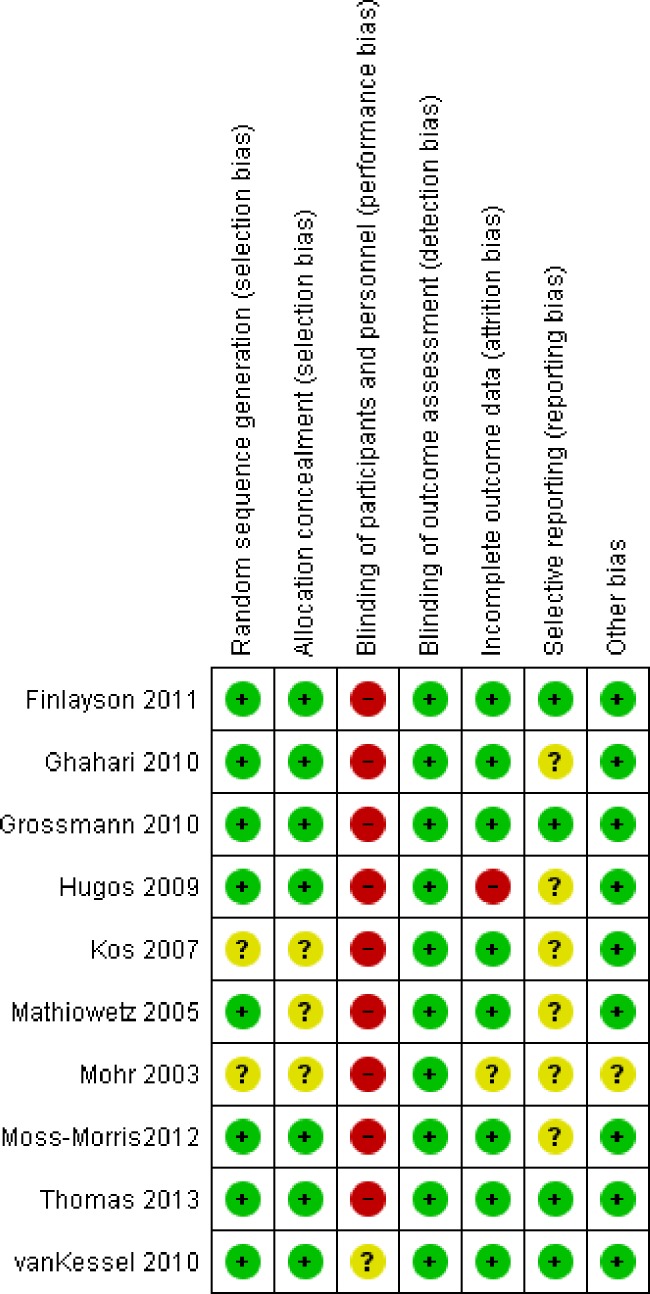
Risk of bias in individual studies. (+) low risk of bias; (?) unclear risk of bias; (-) high risk of bias.

### Synthesis of results

Six studies measured fatigue severity and five measured fatigue impact. Depression was measured by seven studies; while data for quality of life were only available for two studies, so we decided against a quantitative synthesis. Not all studies provided all data necessary to be included in the meta-analysis. Fatigue severity as well as depression data were available from six studies; fatigue impact data were available from four studies. Among further endpoints were anxiety, QUALYs, and personal wellbeing. These were only measured by single studies and will therefore not be discussed further in this review.

**Fig 4:**
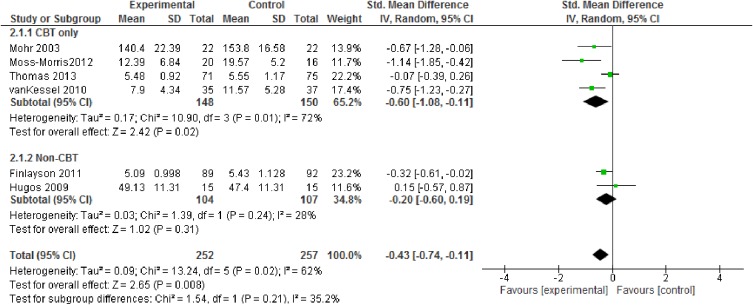
Fatigue severity (subgroups: CBT vs. non-CBT approaches).

Pooled weighted mean difference for fatigue severity (Fig 4) with a pooled sample of n = 509 in six studies was -0.43 (95% CI -0.74 to -0.11; p = 0.008, high quality evidence). CBT-based approaches resulted in more pronounced treatment effects.

**Fig 5:**
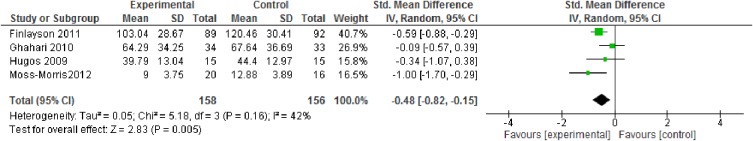
Fatigue impact.

For fatigue impact, the pooled weighted mean difference was -0.48 (95%CI -0.82 to -0.15; p = 0.005, high quality evidence) in four studies with 314 participants (Fig 5).

**Fig 6:**
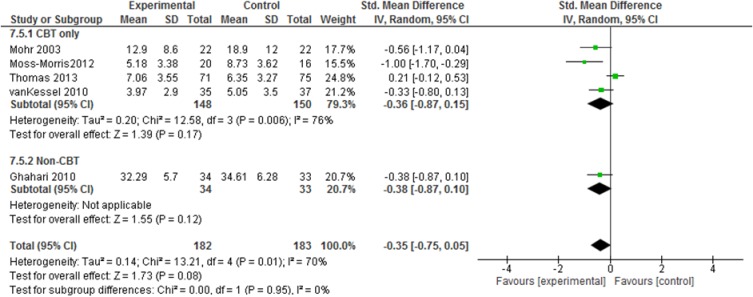
Depression (CBT vs. non-CBT approaches).

Depression data were available for six studies with 365 participants showing no significant effect directly after the intervention with a pooled mean difference of -0.35 (95% CI -0.75 to 0.05; p = 0.08, high quality evidence; Fig 6).

We detected clinical heterogeneity between studies e.g. in terms of interventions, study populations and follow-up periods. Also, most analyses showed marked statistical heterogeneity. Therefore, random effects models were used for meta-analyses.

Analyses indicated a larger effect of CBT-based approaches than other fatigue management programs for both fatigue severity and depression (Figs 4 & 6). For fatigue impact, we decided against calculating subgroup analyses as there were not enough reported data.

**Fig 7:**
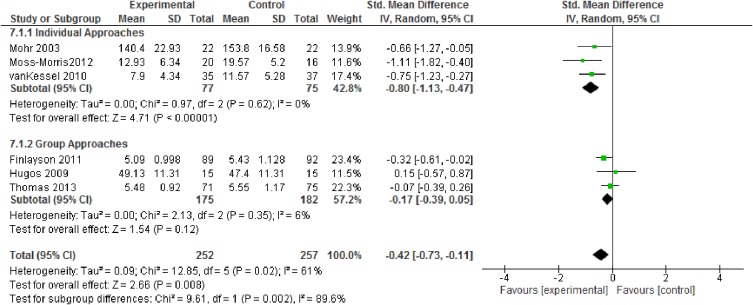
Fatigue severity (subgroups: individual vs. group setting).

**Fig 8:**
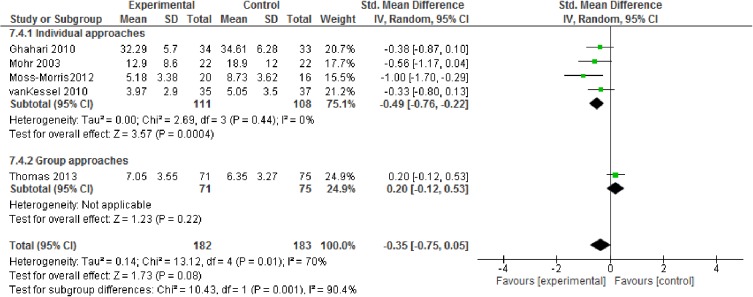
Depression (subgroups: individual vs. group setting).

Furthermore, we analysed the studies that use a face-to-face approach. For fatigue severity and for depression, pooled effect sizes indicated larger effects of individual approaches compared to the group settings (Figs 7 & 8). Again, we decided against analysing subgroup data for fatigue impact due to a lack of data.

## Discussion

Fatigue has been identified as major disabling factor in patients with MS. Accordingly, a number of different pharmacological and non-pharmacological interventions have been developed. This systematic literature review has identified 10 RCTs on educational interventions for MS-related fatigue. Based on the findings of this review, educational interventions seem effective in reducing patient-reported fatigue in the short-term (up to 6 months). There is also some indication that fatigue levels may be reduced over a longer period of time. Follow-up study data from two studies showed sustained reduced fatigue levels after one year [33; 34]. However, more research is warranted.

As usually the case with educational interventions, all included interventions were multi-modal or “complex” interventions comprising different components [35] as e.g. provision of information, interaction with peers, practice of acquired techniques and homework. Also, interventions varied markedly, e.g. in mode of delivery and/or duration. This poses a number of challenges for the synthesis of these interventions. Still, we decided to perform meta-analyses as interventions seemed sufficiently homogeneous in terms of the provided information, setting and the common goal of reducing fatigue. Furthermore, three studies based on the same fatigue management program: Mathiowetz and colleagues tested the “Managing Fatigue” program for the first time while Ghahari et al. and Finlayson et al. used modifications of the same program.

In 2014, Asano & Finlayson published a systematic review comparing the effectiveness of sports interventions, pharmaceutical treatments and educational programs concerning their benefit on MS-related fatigue [14]. The review showed that, of the three intervention types, educational programs had the largest effect in terms of reducing fatigue. In addition to this finding, our review indicates that interventions based on cognitive behavioural therapy seem to be more effective in reducing fatigue compared to other education programs, although this must be taken with caution considering that analyses are based on a small number of studies. This finding is consistent with the assumption that fatigue is, at least partly, caused and perpetuated by negative thoughts and corresponding behaviour [10]. Furthermore, we found some evidence that face-to-face approaches may be superior to group approaches.

The nature of the complex interventions used does not allow a direct comparison of effects of different approaches. Nevertheless, it seems plausible that effectiveness might be increased by combining different methods of intervention provision e.g. online with telephone support sessions or the combination of telephone and face-to-face sessions. Research on mental health conditions suggests, for example, that internet-based interventions tend to be more effective when combined with personal support. Such support is typically provided via e-mail or telephone, and some studies suggest that even minimally trained technicians can be as effective as trained clinicians in their support [36]. Fully automatized support, such as mobile text messages, also appear to be effective across many health conditions and, when offered adjunctively, could boost the effects of internet interventions [37]. Efficient support can also be provided only “on demand” for those who request [38]. Further investigation is needed to examine how different intervention and support elements can be combined to optimize the effects of complex interventions targeting fatigue.

Our findings also suggest that individual approaches may be superior in reducing fatigue severity compared to a group based approach which may be due to closer interaction with the participant and more possibilities of targeting participant-specific problems. On the other hand, group approaches may be easier to implement in a clinical setting as more patients may work with one facilitator at the same time. Furthermore, patients all work together which provides better opportunities for vicarious learning, peer support and modelling. All of this can increase self-efficacy and support behaviour changes. However, no conclusive data on that particular subject are available. Furthermore, the length of the intervention (measured in weeks) and/or the number of sessions might have an impact on their ability to reduce fatigue. The shortest program lasted 4 weeks with 2 hours of program each week without demonstrating a benefit on participants’ fatigue levels [9]. This may be due to shorter practising time since all programs aim to integrate new behavioral strategies into daily life.

The marked reduction of fatigue levels shown by an online CBT intervention by Moss-Morris et al. [25] suggests further exploration of online CBT interventions based on patients’ individual needs and preferences. Evidence from systematic reviews suggests that computer-tailored and web-based interventions that are tailored to suit individual patient characteristics tend to be more effective than non-tailored or “generic” programs. Such evidence mirrors earlier research, which showed that tailored printed health behaviour interventions tend to outperform their non-tailored counterparts [39]. Further research is needed to clarify which forms of tailoring are most effective (e.g., personalization, content-matching, provision of feedback) and by which mechanisms tailoring effects unfold (e.g., enhanced cognitive processing of tailored interventions).

A limitation of the studies included in our review concerns the limited duration of follow-up periods, which lasted from 10 to 52 weeks. To gain insight as to whether participants are able to maintain reduced fatigue levels or if there is a need for ‘booster sessions’ after programs as suggested by some authors, further research with long term outcome measurement is needed.

Concerning limitations of our study, firstly, we only searched the Pubmed and Cochrane databank. Although a single search in Pubmed has shown to be highly sensitive [17], we may still have missed data with this approach. Secondly, our approach of comparing CBT studies to non-CBT studies may be difficult since it is possible to apply principles of CBT without doing pure CBT and some of the non-CBT studies may actually apply CBT techniques as well. So, this may confound the results of our study.

Since MS-related fatigue is reported to be varying individually between patients, all patients should receive the combination of therapy best suited for their individual requirements. Online interventions may be a good compromise between achieving the best possible patient-centred care and actual feasibility of a program in a rehabilitation setting.

This review supports the hypothesis that education programs may be effective in reducing fatigue in patients with MS. Here, CBT-based interventions may be the most effective way to teach patients ways of managing fatigue.

## Supporting information

S1 File Search strategy for PubMed. (TIF)

S2 File Finlayson initially requested teleconf data (Cochrane review, May 25 2014). (DOCX)

S3 File PRISMA checklist. (DOC)

## References

[1] Patti F, Vila C. Symptoms, Prevalence and Impact of Multiple Sclerosis in Younger Patients: A Multinational Survey. Neuroepidemiology 2014;42(4):211–8. 10.1159/000360423 24821493

[2] Braley TJ, Chervin RD. Fatigue in multiple sclerosis: mechanisms, evaluation, and treatment. Sleep. 2010 8;33(8):1061–7. Review. 20815187 10.1093/sleep/33.8.1061 PMC2910465

[3] Mills RJ, Young CA. A medical definition of fatigue in multiple sclerosis. QJM. 2008 1;101(1):49–60. 10.1093/qjmed/hcm122 18194977

[4] Vucic S, Burke D, Kiernan MC. Fatigue in multiple sclerosis: mechanisms and management. Clin Neurophysiol. 2010 6;121(6):809–17. Review. 10.1016/j.clinph.2009.12.013 20100665

[5] Strober LB, Christodoulou C, Benedict RH, Westervelt HJ, Melville P, Scherl WF, et al Unemployment in multiple sclerosis: the contribution of personality and disease. Mult Scler. 2012 5;18(5):647–53. 10.1177/1352458511426735 22183935

[6] Smith MM, Arnett PA. Factors related to employment status changes in individuals with multiple sclerosis. Mult Scler. 2005 10;11(5):602–9. 10.1191/1352458505ms1204oa 16193900

[7] Krupp LB, Serafin DJ, Christodoulou C. Multiple sclerosis-associated fatigue. Expert Rev Neurother. 2010 9;10(9):1437–47. Review. 10.1586/ern.10.99 20819014

[8] Bol Y, Duits AA, Hupperts RM, Vlaeyen JW, Verhey FR. The psychology of fatigue in patients with multiple sclerosis: a review. J Psychosom Res. 2009 1;66(1):3–11. 10.1016/j.jpsychores.2008.05.003 19073287

[9] Kos D, Duportail M, D'hooghe M, Nagels G, Kerckhofs E. Multidisciplinary fatigue management programme in multiple sclerosis: a randomized clinical trial. Mult Scler. 2007 9;13(8):996–1003. 10.1177/1352458507078392 17623738

[10] Knoop H, van Kessel K, Moss-Morris R. Which cognitions and behaviours mediate the positive effect of cognitive behavioural therapy on fatigue in patients with multiple sclerosis? Psychol Med. 2012 1;42(1):205–13. 10.1017/S0033291711000924 21672300

[11] Amtmann D, Bamer AM, Noonan V, Lang N, Kim J, Cook KF. Comparison of the psychometric properties of two fatigue scales in multiple sclerosis. Rehabil Psychol. 2012 5;57(2):159–66 10.1037/a0027890 22686554 PMC3422656

[12] Möller F, Poettgen J, Broemel F, Neuhaus A, Daumer M, Heesen C. HAGIL (Hamburg Vigil Study): a randomized placebo-controlled double-blind study with modafinil for treatment of fatigue in patients with multiple sclerosis. Mult Scler. 2011 8;17(8):1002–9. 10.1177/1352458511402410 21561959

[13] Heine M, Rietberg M, Van Wegen E, Van de Port I, Kwakkel E. Exercise therapy for fatigue in multiple sclerosis. The Cochrany Library 7 2012 CD009956 10.1002/14651858.CD009956.pub2 PMC9554249 26358158

[14] Asano ML, Finlayson M. Meta-analysis of three different types of fatigue management interventions for people with multiple sclerosis: exercise, education, and medication. Mult Scler Int 2014 10.1155/2014/798285 PMC4052049 24963407

[15] Higgins JPT, Altmann DG, Goetzsche PC. “The Cochrane Collaboration’s tool for assessing risk of bias in controlled trials.” British Medical Journal, Vol. 343Article-ID d5928, 2011 10.1136/bmj.d5928 PMC3196245 22008217

[16] Guyatt G, Oxman AD, Akl EA, Kunz R, Vist G, Brozek J, et al. GRADE guidelines: 1. Introduction-GRADE evidence profiles and summary of findings. 10.1016/j.jclinepi.2010.04.026 21195583

[17] Katchamart W, Faulkner A, Feldman B, Tomlinson G, Bombardier C. PubMed had a higher sensitivity then Ovid-MEDLINE in the search for systemtaic reviews. J Clin Epidemiol. 2011 4;64(4):383–94. 20926257 10.1016/j.jclinepi.2010.06.004

[18] Fuhrer MJ. Overview of clinical trials in medical rehabilitation: impetuses, challenges, and needed future directions. Am J Phys Med Rehabil. 2003 10;82(10):S8–15. Review 14502033 10.1097/01.PHM.0000086995.80644.D7

[19] Review Manager (RevMan) [Computer program]. Version Version 5.3. Copenhagen: The Nordic Cochrane Centre, The Cochrane Collaboration, 2014.)

[20] Finlayson M, Preissner K, Cho C, Plow M. Randomized trial of a teleconference-delivered fatigue management program for people with multiple sclerosis. Mult Scler. 2011 9;17(9):1130–40. 10.1177/1352458511404272 21561960

[21] Ghahari S, Leigh Packer T, Passmore AE. Effectiveness of an online fatigue self-management programme for people with chronic neurological conditions: a randomized controlled trial. Clin Rehabil. 2010 8;24(8):727–44. 10.1177/0269215509360648 20543022

[22] Grossman P, Kappos L, Gensicke H, D'Souza M, Mohr DC, Penner IK, et al MS quality of life, depression, and fatigue improve after mindfulness training: a randomized trial. Neurology. 2010 9 28;75(13):1141–9. 10.1212/WNL.0b013e3181f4d80d 20876468 PMC3463050

[23] Hugos CL, Copperman LF, Fuller BE, Yadav V, Lovera J, Bourdette DN. Clinical trial of a formal group fatigue program in multiple sclerosis. Mult Scler. 2010 6;16(6):724–32. 10.1177/1352458510364536 20375125

[24] Mathiowetz VG, Finlayson ML, Matuska KM, Chen HY, Luo P. Randomized controlled trial of an energy conservation course for persons with multiple sclerosis.Mult Scler. 2005 10;11(5):592–601. 10.1191/1352458505ms1198oa 16193899

[25] Moss-Morris R, McCrone P, Yardley L, van Kessel K, Wills G, Dennison L. A pilot randomised controlled trial of an Internet-based cognitive behavioural therapy self-management programme (MS Invigor8) for multiple sclerosis fatigue. Behav Res Ther. 2012 6;50(6):415–21. 10.1016/j.brat.2012.03.001 22516321

[26] Thomas S, Thomas P, Hillier C. A pragmatic parallel arm multi-centre randomised controlled trial to assess the effectiveness and cost-effectiveness of a group-based fatigue management programme (FACETS) for people with multiple sclerosis. J Neurol Neurosurg Psychiatry 2013 10;84(10):1092–9. 10.1136/jnnp-2012-303816 23695501 PMC3786656

[27] Van Kessel K, Moss-Morris R, Willoughby E, Chalder T, Johnson MH, Robinson E. A randomized controlled trial of cognitive behavior therapy for multiple sclerosis fatigue. Psychosom Med. 2008 2;70(2):205–13. 10.1097/PSY.0b013e3181643065 18256342

[28] Mohr DC, Hart SL, Goldberg A. Effects of treatment for depression on fatigue in multiple sclerosis. Psychosom Med. 2003 Jul-Aug;65(4):542–7. 12883103 10.1097/01.psy.0000074757.11682.96

[29] Fisk JD1, Ritvo PG, Ross L, Haase DA, Marrie TJ, Schlech WF. Measuring the functional impact of fatigue: initial validation of the fatigue impact scale. Clin Infect Dis. 1994 1;18 Suppl 1:S79–83. 8148458 10.1093/clinids/18.supplement_1.s79

[30] Kos D, Kerckhofs E, Carrea I, Verza R, Ramos M, Jansa J. Evaluation of the Modified Fatigue Impact Scale in four different European countries. Mult Scler. 2005 2;11(1):76–80. 10.1191/1352458505ms1117oa 15732270

[31] Krupp LB1, LaRocca NG, Muir-Nash J, Steinberg AD. The fatigue severity scale. Application to patients with multiple sclerosis and systemic lupus erythematosus. Arch Neurol. 1989 10;46(10):1121–3. 2803071 10.1001/archneur.1989.00520460115022

[32] Chalder T, Berelowitz G, Pawlikowska T, Watts L, Wessely S, Wright D, et al Development of a fatigue scale. J Psychosom Res. 1993;37(2):147–53. 8463991 10.1016/0022-3999(93)90081-p

[33] Mathiowetz VG, Matuska KM, Finlayson ML, Luo P, Chen HY. One-year follow-up to a randomized controlled trial of an energy conservation course for persons with multiple sclerosis. Int J Rehabil Res. 2007 12;30(4):305–13. 10.1097/MRR.0b013e3282f14434 17975450

[34] Thomas PW, Thomas S1, Kersten P, Jones R, Slingsby V, Nock A, et al One year follow-up of a pragmatic multi-centre randomised controlled trial of a group-based fatigue management programme (FACETS) for people with multiple sclerosis. BMC Neurol. 2014 5 19;14:109 10.1186/1471-2377-14-109 24886398 PMC4046846

[35] Craig P, Dieppe P, Macintyre S, Michie S, Nazareth I, Petticrew M; Medical Research Council Guidance. Developing and evaluating complex interventions: the new Medical Research Council guidance. BMJ. 2008 9 29;337:a1655 10.1136/bmj.a1655 18824488 PMC2769032

[36] Titov N, Andrews G, Davies M, McIntyre K, Robinson E, & Solley K. Internet treatment for depression: a randomized controlled trial comparing clinician vs. technician assistance. PLoS One. 2010 6 8;5(6):e10939 10.1371/journal.pone.0010939 20544030 PMC2882336

[37] Hall AK, Cole-Lewis H, Bernhardt JM. Mobile text messaging for health: a systematic review of reviews. Annual review of public health 36 (2015): 393 10.1146/annurev-publhealth-031914-122855 25785892 PMC4406229

[38] Lustria ML A., Cortese J, Noar SM, Glueckauf RL. Computer-tailored health interventions delivered over the Web: review and analysis of key components. Patient education and counseling 74, no. 2 (2009): 156–173. 18947966 10.1016/j.pec.2008.08.023

[39] Noar S. M., Benac C. N., Harris M. S. (2007). Does tailoring matter? Meta-analytic review of tailored print health behavior change interventions. Psychological bulletin, 133(4), 673 10.1037/0033-2909.133.4.673 17592961

