# Supplementary figures and images for: Patient education for people with multiple sclerosis-associated fatigue: A systematic review
Source: PLoS One. 2017 Mar 7;12(3):e0173025. doi: 10.1371/journal.pone.0173025 (PMC5340368; doi:10.1371/journal.pone.0173025)

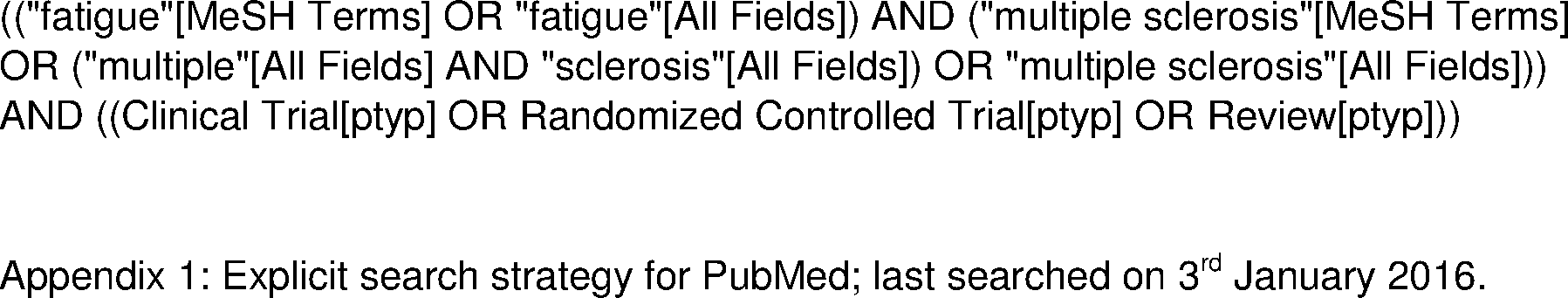

Supplement: S1 File — (TIF) [file pone.0173025.s001.tif]
