# Supplementary material for: Patient education for people with multiple sclerosis-associated fatigue: A systematic review
Source: PLoS One. 2017 Mar 7;12(3):e0173025. doi: 10.1371/journal.pone.0173025 (PMC5340368; doi:10.1371/journal.pone.0173025)
Supplement: S2 File — (DOCX) [file pone.0173025.s002.docx]

**Requested Data – Cochrane Review from: Finlayson, M., Preissner, K., Cho, C., & Plow, M*.* (2011). Randomized trial of a teleconference-delivered fatigue management program for people with multiple sclerosis. *Multiple Sclerosis*, 17 (9), 1130-1140. doi: 10.1177/1352458511404272**

| **FSS average scores** | | | | |
| --- | --- | --- | --- | --- |
| **Time point** | **Delayed entry group** | | **Immediate entry group** | |
|  | **Mean** | **SD** | **Mean** | **SD** |
| **1st pretest for delayed entry group only** | 5.54 | 1.040 |  |  |
| **Immediately pre-intervention** | 5.43 | 1.128 | 5.42 | 0.959 |
| **Post-intervention** | 5.16 | 1.078 | 5.09 | 0.998 |
| **6 weeks post-intervention** | 5.29 | 1.021 | 5.09 | 1.045 |
| **3 months post-intervention** | 5.12 | 1.211 | 4.97 | 1.180 |
| **6 months post-intervention** | 5.29 | 1.142 | 4.99 | 1.142 |
| **FIS total scores** | | | | |
| **Time point** | **Delayed entry group** | | **Immediate entry group** | |
|  | **Mean** | **SD** | **Mean** | **SD** |
| **1st pretest for delayed entry group only** | 124.78 | 27.861 |  |  |
| **Immediately pre-intervention** | 120.46 | 30.406 | 120.19 | 31.067 |
| **Post-intervention** | 110.11 | 29.788 | 103.04 | 28.673 |
| **6 weeks post-intervention** | 106.74 | 29.185 | 101.19 | 28.645 |
| **3 months post-intervention** | 106.75 | 32.771 | 95.33 | 32.120 |
| **6 months post-intervention** | 106.00 | 32.820 | 98.21 | 30.564 |
| **SF36 PHC scores** | | | | |
| **Time point** | **Delayed entry group** | | **Immediate entry group** | |
|  | **Mean** | **SD** | **Mean** | **SD** |
| **1st pretest for delayed entry group only** | 32.14 | 9.729 |  |  |
| **Immediately pre-intervention** | 32.19 | 9.413 | 33.88 | 9.675 |
| **Post-intervention** | 33.84 | 10.286 | 36.15 | 9.854 |
| **6 weeks post-intervention** | 34.69 | 10.019 | 35.95 | 10.155 |
| **3 months post-intervention** | 34.49 | 10.462 | 36.31 | 10.651 |
| **6 months post-intervention** | 34.07 | 10.437 | 36.36 | 10.118 |
| **SF36 PHC scores** | | | | |
| **Time point** | **Delayed entry group** | | **Immediate entry group** | |
|  | **Mean** | **SD** | **Mean** | **SD** |
| **1st pretest for delayed entry group only** | 45.93 | 12.038 |  |  |
| **Immediately pre-intervention** | 46.70 | 11.506 | 44.76 | 12.459 |
| **Post-intervention** | 50.18 | 10.420 | 49.48 | 11.948 |
| **6 weeks post-intervention** | 50.25 | 10.680 | 50.20 | 12.511 |
| **3 months post-intervention** | 48.82 | 11.512 | 50.32 | 10.716 |
| **6 months post-intervention** | 49.08 | 12.857 | 49.51 | 11.399 |
